# Editorial: Ferroptosis, cuproptosis, and triaptosis: unveiling pathways and translational prospects

**DOI:** 10.3389/fcell.2026.1778698

**Published:** 2026-02-13

**Authors:** Jiewei Huang, Yangchun Xie, Fu-Li Xiang, Junqi Huang

**Affiliations:** 1 Key Laboratory of Regenerative Medicine of Ministry of Education, Institute of Aging and Regenerative Medicine, Department of Developmental and Regenerative Medicine, College of Life Science and Technology, Jinan University, Guangzhou, China; 2 Department of Oncology, The Second Xiangya Hospital, Central South University, Changsha, Hunan, China; 3 Institute of Precision Medicine, First Affiliated Hospital of Sun Yat-sen University, Guangzhou, China; 4 NHC Key Laboratory of Assisted Circulation and Vascular Diseases, Sun Yat-sen University, Guangzhou, China

**Keywords:** programmed cell death, ferroptosis, cuproptosis, triaptosis, pathways, translation

## Introduction

Programmed cell death (PCD) is a fundamental feature of multicellular life, governing development, tissue homeostasis, ageing, and disease progression (Newton et al., 2024). While apoptosis, necroptosis, and pyroptosis have long dominated the conceptual framework of PCDs, the past decade has witnessed the rapid emergence of non-canonical modalities shaped by metal- and element-associated metabolic and redox perturbations. Among these, ferroptosis and cuproptosis have established themselves as mechanistically distinct and biologically consequential pathways, while triaptosis has recently been proposed as an oxidative stress-linked form of PCD associated with endosomal dysfunction (Dixon et al., 2012; Tsvetkov et al., 2022; Swamynathan et al., 2024). Mechanistically, ferroptosis is driven by iron-dependent lipid peroxidation and cuproptosis by copper-triggered mitochondrial proteotoxicity, whereas triaptosis is distinctively characterized by PI(3)P-dependent endosomal stress. Together, these modalities highlight a growing appreciation that metal ion homeostasis and redox balance are not merely modifiers of cell metabolism, but central determinants of regulated cellular demise.

This Research Topic, “*Ferroptosis, Cuproptosis, and Triaptosis: Unveiling Pathways and Translational Prospects*” aims to integrate mechanistic discoveries with disease relevance and translational potential across these emerging forms of PCDs. A total of 14 articles are included, comprising 7 original research articles, 6 review articles, and 1 mini-review. Collectively, these articles reflect both the maturity of ferroptosis research and the expanding conceptual landscape of cuproptosis research and oxidative stress-driven cell death pathways.

### Advances in ferroptosis mechanisms and disease contexts

Ferroptosis remains the most extensively explored modality represented in this Research Topic, with contributions spanning both oncology and non-oncology disease contexts, including cancer, fibrotic disorders, and neurodegeneration. The collected studies address ferroptosis across molecular regulation, disease modeling, and therapeutic exploitation, underscoring its broad biological and pathological relevance.

Several original research articles expand the mechanistic boundaries of ferroptosis. For example, Qiao et al. demonstrate that nuclear F-actin assembly is dynamically regulated by intracellular pH during ferroptosis, identifying cytoskeletal remodeling as an underappreciated regulatory layer of the ferroptotic response. This work highlights that structural cellular components and the physicochemical environment, including pH homeostasis, actively shape ferroptotic signaling. Song et al. show that linoleic acid metabolite 13-HODE acts as a biphasic modulator of ferroptosis in granulosa cells, suggesting that metabolic intermediates can both promote and inhibit lipid peroxidation-driven cell death in ovarian follicular atresia.

In oncology-focused studies, Battaglia et al. show that ovarian cancer cells expel iron-rich ferritin via CD63-mediated exosomes to evade ferroptotic stress, identifying iron export as a selectable trait for ferroptosis resistance. In parallel, Zhou and Wang show that targeting NF-κB signaling enhances ferroptosis-based therapeutic strategies in hepatocellular carcinoma, highlighting how classical oncogenic pathways intersect with redox-regulated cell death. Another study by Su et al. highlights the PI3K/Akt signaling pathway as a promising regulator of ferroptosis in cancer, indicating that modulating this axis can influence redox balance and therapeutic susceptibility. Additionally, Wang et al. demonstrate that the synergistic effects of perillaldehyde with ferroptosis inducers in gastric cancer exemplifies how small-molecule combinations can fine-tune ferroptotic sensitivity for therapeutic benefit.

In non-oncology settings, ferroptotic signaling is increasingly implicated in diverse pathological processes. Li et al. show that ferrostatin-1 can alleviate acute sepsis-induced cardiomyopathy by inhibiting neutrophil infiltration and modulating the chemokine axis, highlighting ferroptosis inhibition as a cardioprotective strategy. Fan et al. show that ferroptotic signaling contributes to pulmonary fibrosis and discuss traditional Chinese medicine-driven therapeutic approaches in this context, emphasizing macrophage polarization and fibroblast proliferation as linked to ferroptotic stress. Tang et al. review current evidence linking ferroptosis to neurodegenerative disease progression and highlight potential intervention strategies, including the modulatory effects of exercise. Zhang et al. systematically review how stem cells and their derivatives can modulate ferroptosis in neurodegenerative disease models, outlining regenerative strategies that mitigate iron-dependent oxidative damage. You et al. review that ferroptosis contributes to gestational and hepatic disease pathogenesis through dysregulated iron handling, oxidative stress, and altered lipid metabolism. Together, these articles reinforce ferroptosis as a broadly relevant pathological mechanism rather than a cancer-restricted phenomenon.

### Expanding the scope: cuproptosis and metal-dependent cell death

Cuproptosis represents a newer addition to the PCD repertoire, distinguished by copper-dependent disruption of mitochondrial metabolism and protein lipoylation (Tsvetkov et al., 2022). Although still in its early stages, the inclusion of cuproptosis-focused work in this Research Topic reflects growing interest in copper as a biologically active determinant of cell fate.

Shen et al. review current evidence linking copper imbalance to mitochondrial dysfunction and cardiomyocyte death in myocardial infarction, extending the relevance of cuproptosis beyond oncology into cardiovascular disease. A mini review by Zheng et al. examine the interplay between ferroptosis and cuproptosis in periodontitis, proposing that iron- and copper-dependent death pathways may converge within inflammatory microenvironments to shape tissue damage and disease progression. A clinical-oriented study by Chen et al. perform a comprehensive machine learning-based analysis of cuproptosis- and hypoxia-associated genes in pulmonary arterial hypertension (PAH), identifying AHR, FAS, and FGF2 as key diagnostic markers and linking cuproptosis gene expression patterns to immune infiltration profiles in PAH. Together, these studies underscore the emerging relevance of cuproptosis in diseases, linking copper-dependent cell death to both pathology and diagnostic potential.

### Triaptosis: an emerging concept at the intersection of ROS and endosomal stress

Triaptosis is introduced in this Research Topic as a proposed oxidative stress-driven form of regulated cell death linked to endosomal dysfunction (Swamynathan et al., 2024). Although its molecular framework is less established than that of ferroptosis or cuproptosis, its inclusion highlights an important conceptual advance: dysregulated redox signaling can engage PI(3)P-dependent endosomal pathways to actively shape cell death outcomes. The discussion of triaptosis in this Research Topic is intentionally exploratory, aimed at stimulating rigorous mechanistic and genetic investigation. As with ferroptosis in its early stages, future progress in triaptosis research will depend on the identification of defining molecular markers, core regulators, and physiological or pathological contexts in which this process operates (Qiao et al., 2025).

### Future directions and outstanding challenges

Several overarching themes emerge from this Research Topic, highlighting key priorities for future research. First, mechanistic precision remains essential. For ferroptosis and cuproptosis, efforts should distinguish core execution machinery from context-dependent modulators, while for triaptosis, first-in-class chemical inhibitors and deeper molecular insights are needed. Second, cross-talk between death pathways warrants further exploration, as ferroptosis, cuproptosis, and oxidative stress-associated programs may intersect or compete depending on metabolic state, metal availability, and cellular context, especially in diseases with metabolic dysregulation and chronic inflammation. Third, translational integration remains a central challenge. Although ferroptosis modulators approach clinical relevance, reliable biomarkers, patient stratification, and delivery strategies remain limiting factors. Comparable translational frameworks should be developed early for cuproptosis- and triaptosis-targeted interventions to avoid repeating prior bottlenecks. Finally, metallo-redox biology emerges as a unifying concept: iron and copper are not merely toxic catalysts but integral components of cellular signaling networks. Future studies leveraging metallomics, spatial imaging, and systems-level approaches will be essential to decode how metal ions orchestrate cell fate in health and disease.

## Concluding remarks

The articles collected in this Research Topic reflect both consolidation and expansion within the field of PCD. By integrating established knowledge of ferroptosis with emerging insights into cuproptosis and triaptosis, this Research Topic highlights the evolving complexity of cell death biology and its translational promise. We thank all authors and reviewers for their valuable contributions and hope that this Research Topic will stimulate continued innovation at the intersection of PCDs, metal/ion homeostasis, and redox biology.
